# A short review on the features of the non-obese diabetic Goto-Kakizaki rat intestine

**DOI:** 10.1590/1414-431X2022e11910

**Published:** 2022-08-22

**Authors:** G.M. Gimenes, G.O. Santana, M.V.M. Scervino, R. Curi, J.N.B. Pereira

**Affiliations:** 1 Programa de Pós-Graduação Interdisciplinar em Ciências da Saúde, Universidade Cruzeiro do Sul, São Paulo, SP, Brasil; 2 Centro Bioindustrial, Instituto Butantan, São Paulo, SP, Brasil; 3 Laboratório Estratégico de Diagnóstico Molecular, Instituto Butantan, São Paulo, SP, Brasil

**Keywords:** Gastrointestinal tract, Type 2 diabetes, GK rats, Intestinal microbiota, Diabetes treatment

## Abstract

The Goto-Kakizaki (GK) rat is a non-obese experimental model of type 2 diabetes mellitus (T2DM) that allows researchers to monitor diabetes-induced changes without jeopardizing the effects of obesity. This rat strain exhibits notable gastrointestinal features associated with T2DM, such as marked alterations in intestinal morphology, reduced intestinal motility, slow transit, and modified microbiota compared to Wistar rats. The primary treatments for diabetic patients include administration of hypoglycemic agents and insulin, and lifestyle changes. Emerging procedures, including alternative therapies, metabolic surgeries, and modulation of the intestinal microbiota composition, have been shown to improve the diabetic state of GK rats. This review describes the morpho-physiological diabetic-associated features of the gastrointestinal tract (GIT) of GK rats. We also describe promising strategies, e.g., metabolic surgery and modulation of gut microbiota composition, used to target the GIT of this animal model to improve the diabetic state.

## Introduction

It is predicted that 783 million people will be diagnosed with diabetes by 2045. Among the types of DM, we highlight type 2 diabetes mellitus (T2DM), which currently accounts for 90% of diabetes cases (1). This chronic multifactorial disease is caused by insulin resistance (IR) and results in hyperglycemia. It is well known that obesity is associated with low-grade systemic inflammation, a risk factor for developing IR and T2DM. At the molecular level, increased plasma cytokine levels promote serine phosphorylation of insulin receptors, impairing insulin signaling and attenuating insulin sensitivity and response (i.e., an IR state).

Around 80% of adult T2DM patients worldwide are considered overweight or obese, and reducing body weight improves blood glucose levels (2). In addition to obesity, periodontal disease, obstructive pulmonary disease, arthritis, and muscular dystrophy (3-5) have also been linked to T2DM development (6). Despite the well-established association between obesity and IR, 10-15% of people with this disease are not obese (7). Hartmann et al. (8) combined data from two German databases to compare lean and obese individuals with type 2 diabetes. The results showed that non-obese T2DM patients have higher mortality rates and hypoglycemic events. They also reported that smoking and alcohol consumption and chronic kidney disease are increased among lean T2DM patients.

The Goto-Kakizaki (GK) rat is a non-obese animal model that spontaneously develops type 2 diabetes early in life. This animal strain was developed at Tohoku University in Sendai, Japan, in 1975 by Goto and Kakizaki through successive inbreeding of non-diabetic Wistar rats with mild glucose intolerance. After five generations, the deliberate, repeated selection of rats with impaired glucose tolerance resulted in the establishment of glucose intolerance. In the late 1980s, GK rats were introduced into isogenic reproductive colonies in several countries and made commercially available by Japanese breeders (9,10).

**Figure 1:**
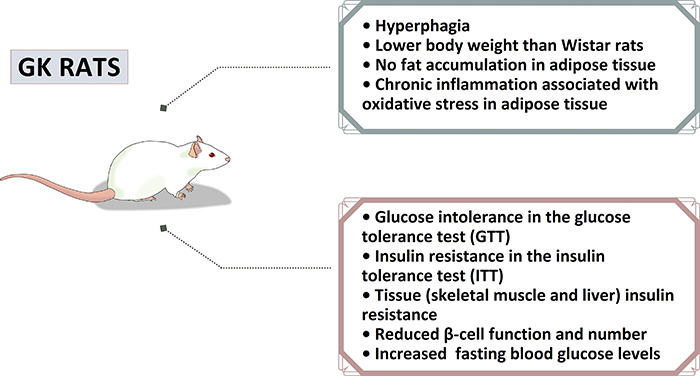
Main features reported in Goto-Kakizaki (GK) rats, a non-obese type 2 diabetes mellitus experimental model.

GK rats exhibit chronic inflammation, reduced pancreatic β-cell function and number, moderate hyperglycemia, impaired glucose-induced insulin secretion, glucose intolerance, and peripheral IR (10,11) (see Figure 1). Since this non-obese T2DM experimental model does not require diet-induced obesity, researchers can study the genetic factors and molecular mechanisms associated with T2DM development without obesity-induced effects.

Matafome et al. (12) studied the adipose tissue of Wistar and GK rats at six weeks and 14 months of age. There was an increase in advanced glycation end products (AGEs), triglycerides, and fibrosis, which worsened in aged GK rats. Additionally, the authors reported attenuated tissue blood irrigation with interstitial hypoxia. Moreover, Rodrigues et al. (13) submitted GK rats to diet restriction and observed reduced lipid peroxidation, oxidative stress markers, and IR and high levels of cholesterol, free fatty acids, triglycerides, and intramyocellular triglyceride content.

Kuwabara et al. (14) compared two experimental models of T2DM: GK rats and high-fat-diet-induced obese rats. The authors reported white adipose tissue (WAT) expansion, increased inflammatory cell infiltration, elevated cytokine concentrations, and fat accumulation in the livers of high-fat-diet-fed animals. On the other hand, the GK group displayed attenuated p-AKT expression in WAT, liver glycogen accumulation, and increased inflammatory cytokine expression in the liver. Our group recently reported intestinal remodeling (e.g., hyperplasia and morphological changes) and inflammation associated with slow intestinal transit in GK rats (15). It is plausible that systemic inflammation and strain-specific intestinal morpho-physiological features reported in GK rats could drive IR pathogenesis and T2DM development in these animals and perhaps in non-obese patients.

The brown adipose tissue (BAT) of GK rats was studied and compared in different diet-induced T2DM models. The authors reported a process of whitening and impaired function in the BAT of 16-week-old GK rats, decreased gene expression of glucose transporter 1, and an increase in expression of genes involved in fatty acid oxidation, BAT metabolism, and leptin serum concentrations. A histological evaluation of BAT indicated reduced cellular density with greater adipocyte area in GK rats (16).

Presently, hypoglycemic agents, insulin administration, and changes in lifestyle are the primary T2DM treatments (17). Metabolic surgeries and modulation of the microbiota composition in the gastrointestinal tract (GIT) recently gained attention as alternative therapies for improving the diabetic state (18,19). The intestinal microbiota contributes to the intestinal barrier and plays essential roles in controlling gut motility, delivering vitamins, supporting host immunity, and modulating gut-brain axis communication (20,21). An imbalance in the microbiota composition and augmented gut microbiota-derived metabolite production (i.e., intestinal dysbiosis) significantly influence the pathogenesis and progression of obesity and T2DM (22,23). Based on these results, the GK rat microbiota has been proposed as a possible therapeutic target (24-26).

This review summarizes the studies describing the intestinal morpho-physiology and microbiota of GK rats. We also discuss the potential of targeting the intestine to treat T2DM since intestinal function is associated with the diabetic state. Understanding the underlying mechanisms involved in these processes will direct future studies on intestine-based therapies to treat T2DM individuals, including non-obese ones.

## Eligibility criteria and main points for study selection

**Figure 2:**
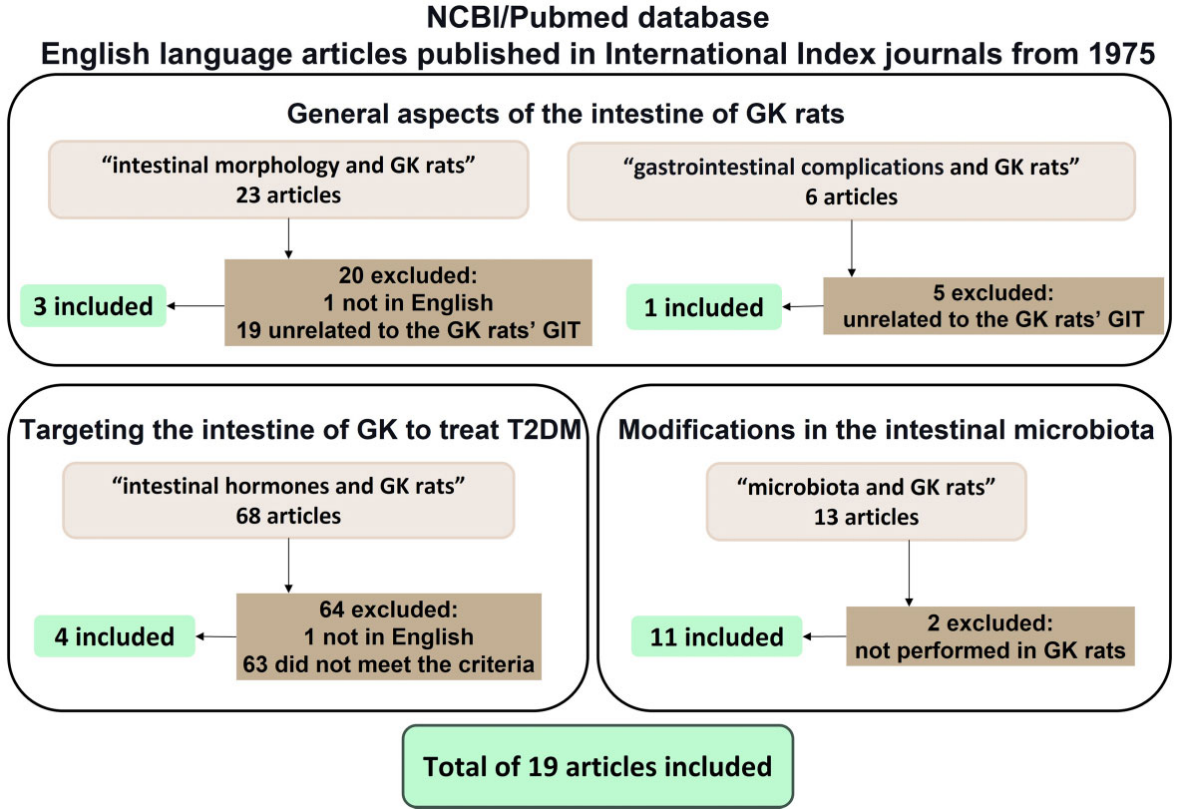
Flow diagrams for selecting articles for each topic. GK: Goto-Kakizaki; T2DM: type 2 diabetes mellitus; GIT: gastrointestinal tract.

We conducted a literature search using the NCBI/PubMed database. English language articles published in international indexed journals since 1975 were considered for selection. Potential articles were identified using a search strategy that considered the title, abstract, and full-text review (Figure 2). Discrepancies about inclusion/exclusion were resolved through discussion or third-party mediation.

The search term “Goto-Kakizaki” was present in 1085 articles. Replacing the search term “Goto-Kakizaki” with “GK” retrieved more articles. The search term “intestine and Goto-Kakizaki” yielded 72 results. In recent years, there has been an increase in publications on both topics. It is important to point out that we applied different criteria for each topic to select the articles presented in this review. These criteria were based on previous research and discussion among the authors. We also screened the references of primary studies to identify other relevant articles.

The morpho-physiology of the GK rat intestine topic was investigated using the search term “intestinal morphology and GK rats”, which returned 23 results. Twenty of these studies were excluded; one was not in English, and 19 were not explicitly related to the GIT of GK rats. Additionally, the search term “gastrointestinal complications and GK rats” retrieved six articles, but five were unrelated to the GK rat intestine.

The search using the term “metabolic surgery and Goto-Kakizaki” retrieved 73 articles. This topic was the most explored issue in the GIT of GK rats. However, four articles were not in English, one was a review, and twelve were unrelated to metabolic surgeries, which resulted in 57 articles on metabolic surgeries in GK rats. This review briefly addresses metabolic surgery in GK rats mentioning some basic concepts (e.g., the theories and general results obtained), but it deserves to be discussed in a dedicated review.

We used the term “intestinal hormones and GK rats” to search for articles that chemically targeted the intestine of GK rats to influence or regulate bowel function and improve the T2DM state. The search yielded 68 articles; one was not written in English and 63 did not meet the criteria; thus, four remained.

The search term “microbiota and GK rats” was used to search for studies about intestinal microbiota modifications in GK rats. This search retrieved 13 articles; two studies were not performed in GK rats, leaving 11 articles for this topic.

## General aspects of the intestine of GK rats

This topic included studies that evaluated the intestinal morphology (three articles) or reported gastrointestinal complications (one article) in this animal model. The selected articles could be further divided into the following subtopics: morpho-physiology, inflammation, enteric nervous system (ENS), and motility.

## Morpho-physiology

Adachi et al. (27) described marked differences in the intestinal morphology and physiology in 10- and 20-week-old GK rats compared to Wistar animals. For example, the height of villi in the small intestine of GK rats was significantly greater than in the respective controls for both age groups. The levels of enzymes involved in the breakdown of carbohydrates into glucose (e.g., isomaltase and sucrase) were also elevated in the GK rat intestine. GK rats also exhibited intestinal hyperplasia, possibly due to the increased expression of transcription factors and proteins involved in cell regeneration, differentiation, and/or proliferation.

Pereira et al. (15) assessed small intestine remodeling in intestine segments of 16-week-old male GK rats. The morphometric analysis indicated increased thickness of the muscle layer in the duodenum, jejunum, and ileum. The GK rats also exhibited significantly greater villi heights in the jejunum and ileum and thicker villi in the duodenum and ileum compared to control animals. Moreover, crypt depth was reduced in the duodenum and ileum and increased in the jejunum of GK rats.

Other studies have reported muscle layer hypertrophy in the large (28) and small intestines (29) of 32-week-old GK rats. One study reported that the villi thickness is increased in the jejunum (29). Significant biomechanical differences were also described, such as smaller opening angles and residual stress-strain values, in GK rats. The residual stress-strain curve was shifted to the left, indicating that the jejunum wall is stiffer than normal. Residual stress and strain forces have been implicated in protecting the mucosa, and alterations in these parameters are linked to remodeling and cell growth (30). The same authors concluded that the viscoelastic properties of the diabetic group impair sensory and motility functions of the intestine. It has also been proposed that the structure and deformation changes may alter the relative positions of the mechanosensitive afferents (31).

## Inflammation

Intestinal inflammation and increased IL-1β content in the duodenum, jejunum, and ileum muscle layers have also been reported in GK rats (15). Upregulated NF-κB expression in portions of the GK rat small intestine compared to control animals has also been observed. These results are not surprising since NF-κB protein transcription factor upregulates the expression of specific inflammatory genes, including IL-1β (15), consequently activating the NF-κB pathway, promoting IκB degradation, and inducing NF-κB nuclear translocation. The authors also reported a positive auto-regulatory loop in the GK intestine that amplifies the inflammatory response and local inflammation. GK rats also exhibit markedly slower intestinal transit compared to control animals. Since IL-1β causes intestinal hypomotility (32), increased expression of IL-1β and NF-κB could then be the cause for the reduced intestinal transit in GK rats.

In addition to inflammation markers, AGE content and AGE receptor (RAGE) expression were measured in the small and large intestines of 32-week-old GK rats. The staining intensities of AGE in epithelial cells from villi and crypts of GK rats were more robust than in Wistar animals in the duodenum (28) and jejunum (29). The intensity of RAGE immunoreactivity was also increased in villi of the jejunum of GK rats. The AGE deposits were mainly detected in epithelial cells, impacting intestinal absorption, enzyme activities, and digestive activities. Increased RAGE immunoreactivity was also reported in myenteric and submucosal neurons from the small and large intestines of 32-week-old GK rats compared to control animals (28).

## ENS and intestinal motility

The GK rat ENS alterations and slow GI transit have been described in other T2DM animal models and associated with diabetic autonomic neuropathy (33). The ENS is a division of the autonomic nervous system composed of two myenteric and submucosal plexuses (34).

The total neuronal populations of myenteric and submucosal plexuses and the total neuronal densities of the myenteric plexus in the duodenum, jejunum, and ileum of 16-week-old GK rats are not significantly different from controls. However, myenteric neuronal and ganglion hypertrophy was observed in the small intestine segments of GK rats. A reduced number of ganglion neurons in the jejunum and ileum of the submucosal plexus was also observed. Moreover, the neuronal and ganglion areas were increased in the myenteric plexus of the duodenum, jejunum, and ileum of GK rats. However, there was no difference in myenteric density, and neuronal subpopulations were not addressed in this study (15).

T2DM induces marked changes in enteric neurons, especially in neuronal subpopulations of the intestine. Pereira et al. (15) reported that the total neuronal population of the submucosal plexus is more susceptible to degenerative changes than the myenteric plexus in GK rats. In this sense, enteric neuronal hypertrophy may be a compensatory mechanism for maintaining intestinal functions in this animal model.

Due to metabolic-inducing factors, such as ENS oxidative stress, resulting from the imbalance between reactive oxygen species (ROS) production and removal, degenerative changes have been implicated in the pathogenesis of diabetic neuropathy and other complications (35,36). Hyperglycemia-related oxidative stress and inflammation are primary inducers of ENS dysfunction and result from significant changes in intestinal motility and secretion activity (37-39).

Increased IL-1β reactivity has been reported in myenteric neurons and glial cells from the small intestines of GK rats compared to Wistar rats (15). A strong reactivity of RAGEs has also been described in the enteric neurons of the small and large intestines of GK rats (28). Notably, myenteric cells (neurons and glial cells) directly contribute to the intestinal inflammatory response via inflammatory mediator production (40).

Neuronal AGE formation and subsequent accumulation would be expected to have structural and functional consequences at the protein level and directly participate in GIT neuropathy development. For example, the neurotransmitter nitric oxide (NO), produced by neuronal nitric oxide synthase (nNOS) in GI nerves, regulates GIT motility but can induce neuronal apoptosis in the presence of AGEs *in vitro*. In this sense, neuronal RAGE accumulation may contribute to the slow GI transit reported in GK rats. Interestingly, Sena et al. (41) reported endothelial dysfunction associated with AGEs, decreased response to NO, oxidative stress, and inflammation, all worsened by methylglyoxal treatment in GK rats.

## Targeting the intestine of GK rats to treat T2DM

The GK rat is the most utilized non-obese experimental model for T2DM studies (42,43). Our literature search revealed that intestinal interventions are the most studied topic related to the GIT of GK rats. In general, these studies sought to identify weight-loss-independent mechanisms involved in the diabetic state.

The most prevalent intestinal intervention in GK rats is metabolic surgery, for which a review should be written. Some surgical procedures are based on the foregut theory, which reduces the contact time of the ingested food with the distal intestine to improve diabetic conditions. On the other hand, the hindgut theory considers hormonal factors, such as glucagon-like peptide-1 (GLP-1) and peptide YY (PYY), that are produced by L cells and abundant in the distal portion of the small intestine (44). The surgical procedures performed on GK rats include the Roux-en-Y gastric bypass, duodenal-jejunal bypass, ileal transposition, and sleeve gastrectomy. Most studies reported that these surgeries improve glucose tolerance, reduce IR, alter plasma hormone (e.g., incretins) levels, and/or impair insulin signaling and response (45-47).

Other GIT-related strategies to treat the diabetic state include modulating the intestine's endocrine function. The administration of intestinal hormones has been evaluated as a therapy for improving the GK rat diabetic condition. For example, administering GLP-1, an insulinotropic hormone, improves glucose tolerance, inhibits intestinal motility, and reduces blood flow in pancreatic islets, duodenum, and colon of GK rats (48,49). GLP-1 and the glucose-dependent insulinotropic peptide (GIP) appear to stimulate insulin secretion. Moreover, upregulated GLP-1 and GIP receptor gene expressions have been reported in the pancreatic islets and the small bowel of GK rats (48).

Some drugs may interact with incretin metabolism. Simonsen et al. (50) evaluated the effect of exendin-4 (GLP-1 receptor agonist) and dipeptidyl peptidase IV inhibitor (DPPIV inhibitor) in 12-week-old GK rats to determine if they could disrupt intestinal growth. The two compounds improved the hyperglycemic condition by lowering HbA1c levels. The exendin-4-treated GK animals exhibited lower body mass, which was abolished after treatment cessation. Additionally, exendin-4 treatment was associated with increased intestinal length and weight, of which the latter was restored following the experimental period. Furthermore, the intestinal cross-sectional area was not detected in the DPPIV treated group.

As already mentioned, GK rats exhibit slow intestinal transit (15). This condition can be alleviated with acute intestinal electrical stimulation (51), which has also been shown to improve glucose intolerance. Moreover, chronic electrical stimulation reduced blood glucose and pancreatic β-cell apoptosis and increased plasma GLP-1 levels (51).

Using GK rats as an experimental model to investigate the treatment of diabetes through metabolic surgeries or the administration of intestinal hormones or plant-derived compounds revealed viable options with optimistic results. In this sense, it appears as though the intestine has a significant association with the diabetic state in GK rats. These observations highlight the need to elucidate the underlying mechanisms of T2DM onset and progression in GK rats.

## Features of GK rat intestinal microbiota

The intestinal microbiota and fecal metabolites of 15-week-old GK rats were evaluated by Peng et al. (24). GK rats displayed decreased alpha and beta diversity values compared to control animals. The predominant genus in GK rats was *Bacteroidates*, *Lactobacillus*, and *Prevotella*, whereas *Bacteroidates* and *Lachnospiraceae* were most prominent in Wistar rats. GK rat fecal metabolites differed significantly from Wistar rats representing potential T2DM biomarkers. Five metabolic pathways are impaired in this animal model, including phenylalanine, tyrosine, and tryptophan biosynthesis, glycerophospholipid metabolism, sphingolipid metabolism, tyrosine metabolism, and steroid hormone biosynthesis.

Kang et al. (25) evaluated the gastric microbiota of 20-week-old GK rats and found that microbiota richness of diabetic and control groups was similar. However, GK rats have less diverse gastric microbiota, which has been implicated in several gastrointestinal diseases. The authors also reported that *Firmicutes* is the most abundant phylum of bacteria in the microbiota of GK rats (96% of the microbial composition *vs* 72.9% in Wistar). They correlated the increased *Firmicutes* to *Bacteroidetes* species ratio with obesity and T2DM. Another observation was that the GK rat gastric microbiota is composed of a higher proportion of chemoheterotrophic bacteria and those that carry out fermentation, potentially perturbing blood glucose levels (25). Due to the strong relationship of intestinal microbiota with intestinal morphology and chronic inflammation (52,53), it has become a target for studies on the genesis of T2DM.

The primary metabolic products of intestinal microbiota digestion of non-absorbable dietary fiber and resistant starches include short-chain fatty acids (SCFAs), such as acetate, propionate, and butyrate (54). Butyrate is a notable SCFA because it antagonizes intestinal inflammation by inhibiting the NF-κB transcription factor, which regulates innate inflammatory immune responses (55) and inhibits interferon-gamma (IFN-γ) signaling. The NF-κB transcription factor also stimulates intestinal regulatory T-cell production and upregulates the expression of peroxisome proliferator-activated receptor γ (PPARγ) to prevent inflammation (56,57). SCFAs also influence intestinal morphology, villi height, and crypt depth, directly impacting nutrient digestion and absorption (56,57). Indeed, increasing SCFA production improves intestinal health (58,59).

The high *Firmicutes* content in the intestine of GK rats inhibits the growth of bacterial phyla that produce SCFAs (24), including *Actinobacteria* and *Proteobacteria* (60). Increased proportions of specific bacteria (e.g., gram negative bacteria such as *Bacteroidetes* and *Proteobacteria*) have been associated with elevated lipopolysaccharide (LPS) production, triggering a chronic inflammatory response, contributing to disease onset and progression (24). While the high microbial content of *Bacteroidetes* and *Proteobacteria* is harmful to the host, others are associated with a better T2DM prognosis. This information highlights the need to identify and characterize the intestinal microbiota at different diabetic stages and under different DM-inducing conditions.

Studies on diabetic patients and high-fat-fed mice reported microbiota modulation by metabolic surgery (23,61-63). In GK rats, the sleeve gastrectomy leads to improved glucose tolerance, accompanied by enrichment of cecal *Prevotella copri*. An improvement in glucose tolerance was observed when the microbiota from the metabolic surgery animals was transferred to non-operated ones (26).

Several studies have focused on using plant-derived therapies, or of “natural origin”, to treat the complications caused by DM or to complement the typical treatments, such as metabolic surgeries. Some active agents improved diabetes by inducing changes in the GIT components, promoting a positive modulation of microbiota composition. We summarized studies that modulate microbiota composition to improve diabetes, including natural substances. Information on these studies (e.g., study length, substance administration, and main findings) is shown in Table 1 (26,60,64-70).

**Table 1:** Studies on interventions in the intestinal microbiota of Goto-Kakizaki (GK) rats.

Reference	Procedure	Study length	Final age	Body weight	Main findings
Qiao et al., 2018 (64)	Supplementation with *Paenibacillus bovis* sp. nov. BD3526	8 weeks	21 weeks old	↓	↓ HbA1C; ↑ microbiota diversity at weeks 2 and 3, which was restored after 6 weeks; ↔ SCFA-producing bacteria.
Zhao et al., 2018 (65)	HFD and liraglutide injection	8 weeks of HFD and 12 weeks of injection	24 weeks old	↓	Improvement of glucose and lipid metabolism; ↑ intestinal microbiota abundance and diversity.
Zhang et al., 2019 (66)	Supplementation with Extract of Ice Plant (IPE)	8 weeks	17 weeks old	↓	Improved glycemic control, pancreatic islet morphology, beta-cell survival, insulin secretion and composition of intestinal microbiota; ↓ glycated serum proteins; ↓ HOMA-IR.
Zhao et al., 2019 (67)	Supplementation with Chinese herbal formula Shenzhu Tiaopi Granule	8 weeks	17 weeks old	↓	↓ Total cholesterol; changes in microbiota by enrichment of *Proteobacteria*.
Zhao et al., 2020 (68)	Supplementation with sea cucumber *Holothuria leucospilota* polysaccharide (HLP)	4 weeks	23 weeks old	↔	Improved glycemic control and lipid levels; ↓ serum insulin, adiponectin, and abnormal insulin signaling, and apoptosis-related molecules; ↑ serum leptin and GLP-1 and insulin signaling and anti-apoptotic genes; ↑ SCFA-producing bacteria and ↓ opportunistic pathogenic bacteria in GK feces; ↑ doses of HLP eliminated damage to the pancreas and colon of diabetic rats.
Péan et al., 2020 (26)	Vertical sleeve gastrectomy (VSG) and transfer of the intestinal microbiota	13 weeks	29 weeks old	↔	Enrichment of *Prevotella copri* improved glucose and bile acid metabolism.
Yu et al., 2020 (60)	Single-anastomosis duodenal jejunal bypass (DJB-sa)	8 weeks	21 weeks old	↔	Improved glycemic control; ↓ fasting serum insulin; ↑ GPL-1, SCFA receptors and SCFA-producing bacteria.
Tan et al., 2021 (69)	Modified jejunoileal bypass (SSJIBL)	6 weeks	14 weeks old	↓	Improved glycemic control. ↓ serum lipids, FFAs and liver injury markers; ↑ GLP-1, TNF-α, IL-6, insulin expression, and proliferation marker. ↑ Firmicutes and Proteobacteria and ↓ of Bacteroidetes in GK feces after surgery.
Zhao et al., 2021 (70)	Supplementation with berberine	8 weeks	15 weeks old	↔	↑ GLP-1 and ↓ HOMA-IR; improvement of pancreatic islet morphology.

↓: decreased; ↑: increased; ↔: no change; HbA1C: glycated hemoglobin; SCFA: short-chain fatty acid; HFD: high-fat diet; IPE: ice plant extract; HOMA-IR: homeostatic model assessment of insulin resistance; HLP: *Holothuria leucospilota* polysaccharide; GLP-1: glucagon-like peptide 1; GK: Goto-Kakizaki; VSG: vertical sleeve gastrectomy; DJB-sa: single-anastomosis duodenal jejunal bypass; SSJIBL: modified jejunoileal bypass; FFA: free fatty acid; TNF-α: tumor necrosis factor-alpha; IL-6: interleukin 6.

## Pinpointing the main issues

**Figure 3:**
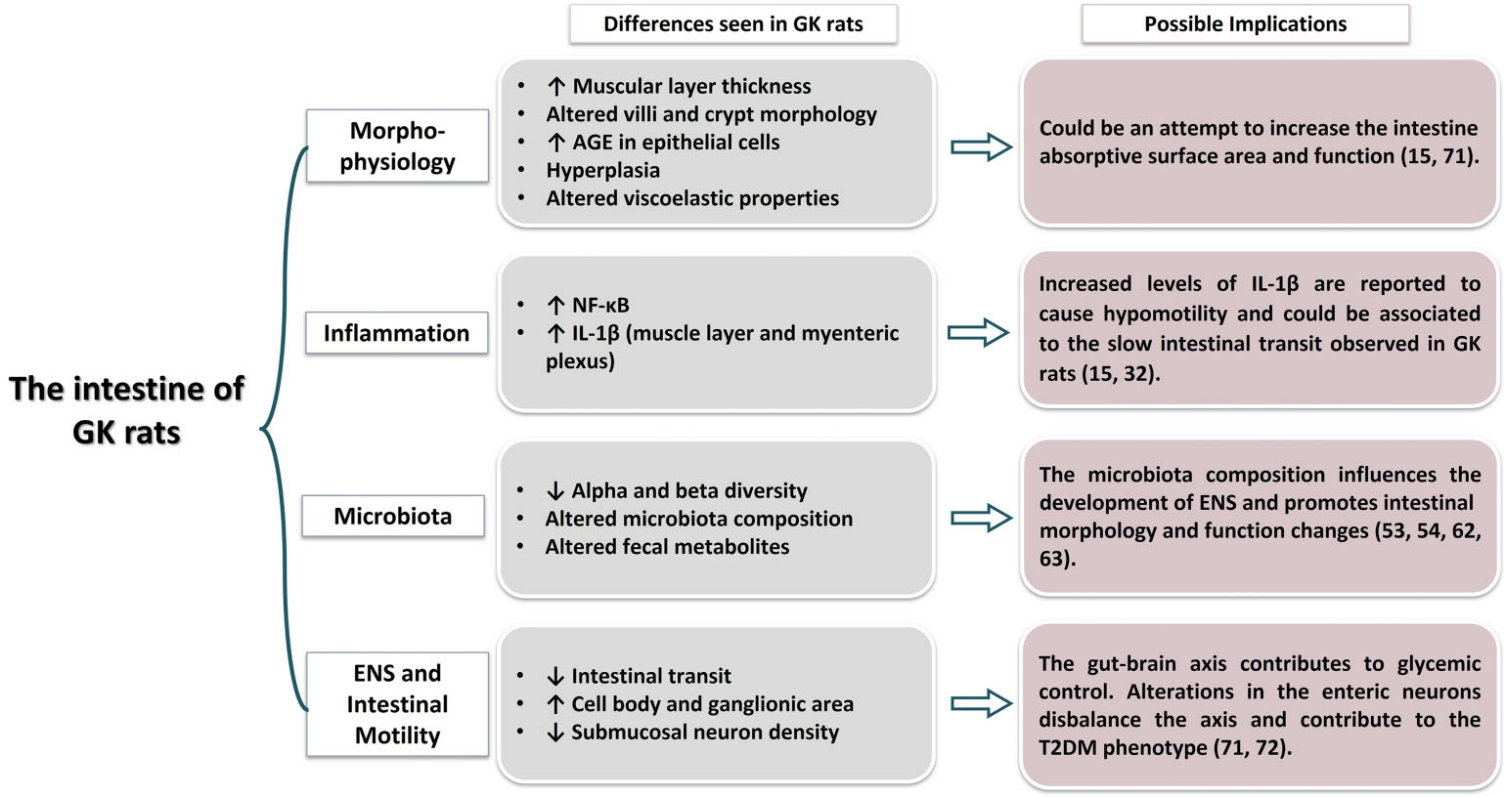
The pinpoint issues of the intestine of Goto-Kakizaki (GK) rats. ENS: enteric nervous system; GIT: gastrointestinal tract; AGE: advanced glycation end-product.

The GIT of GK rats has been extensively used to study strategies to improve the diabetic state. The changes in the intestine of GK rats (Figure 3) discussed in this review were also seen in other experimental models and associated with the pathophysiologic mechanisms for T2DM establishment and or progression that include intestinal inflammation and altered morphology, ENS disruption, gut motility, and microbiota composition.

The intestinal microbiota is associated with the production of metabolites and the synthesis of neurotransmitters, influencing ENS physiology (71,72). Moreover, microbiota composition might influence intestinal morphology (e.g., villi height and crypt depth), digestion, and absorption in GK rats (56,57).

ENS neurons monitor luminal content by modulating intestinal motility and transit, nutrient digestion, and absorption (73). The impaired intestinal motility of GK rats suggests that ENS components, e.g., distribution of neuronal subpopulations, may be structured differently in this animal model. Since the ENS communicates with other systems, such as the immune system and gut-microbiota-brain axis (74,75), dysfunctions in the ENS can contribute to pathological complications, as in diabetes. For instance, the gut-brain axis can control glucose metabolism by an IR-associated efferent hypothalamic signal caused by duodenal hyper-contractility (72,76). Changes in GLP-1 concentrations are notable in the intestine of GK rats due to metabolic surgery or other therapeutic strategies. This observation is not entirely surprising since GLP-1 reduces food intake, controls glycemia, and inhibits intestinal motility (73). Accumulating evidence has shown that the gut contributes to the establishment and prognosis of T2DM in non-obese GK rats.

Regarding the clinical application in T2DM patients, the intestinal interventions (e.g., metabolic surgeries, modulation of gut microbiota composition, and administration of natural products) addressed in this review are valuable strategies for complementing typical DM treatments, including pharmacological intervention and improved lifestyle with a balanced diet and regular physical activity. Conventional approaches and gut function manipulation can help physicians achieve better glycemic and metabolic control of T2DM patients.

## References

[1] Federation. ID (2021). Journal of Experimental Biology.

[2] Schwartz SS, Kohl BA (2010). Glycemic control and weight reduction without causing
hypoglycemia: The case for continued safe aggressive care of patients with
type 2 diabetes mellitus and avoidance of therapeutic
inertia. Mayo Clin Proc.

[3] Demmer RT, Jacobs DR Desvarieux M (2008). Periodontal disease and incident type 2 diabetes: results from
the First National Health and Nutrition Examination Survey and its
epidemiologic follow-up study. Diabetes Care.

[B04] McNeely MJ, Boyko EJ (2004). Type 2 diabetes prevalence in Asian Americans: results of a
naitonal health survey. Diabetes Care.

[5] Tiengo A, Fadini GP, Avogaro A (2008). The metabolic syndrome, diabetes and lung
dysfunction. Diabetes Metab.

[6] Wondmkun YT (2020). Obesity, insulin resistance, and type 2 diabetes: Associations
and therapeutic implications. Diabetes Metab Syndr Obes.

[7] Goto A, Noda M, Goto M, Yasuda K, Mizoue T, Yamaji T (2018). Predictive performance of a genetic risk score using 11
susceptibility alleles for the incidence of Type 2 diabetes in a general
Japanese population: a nested case-control study. Diabet Med.

[8] Hartmann B, Lanzinger S, Bramlage P, Groß F, Danne T, Wagner S (2017). Lean diabetes in middle-aged adults: a joint analysis of the
German DIVE and DPV registries. PLoS One.

[9] Goto Y, Kakizaki M, Masaki N (1975). Spontaneous diabetes produced by selective breeding of normal
wistar rats. Proc Jpn Acad.

[10] Ahmed MS, Pelletier J, Leumann H, Gu HF, Östenson CG (2015). Expression of protein kinase C isoforms in pancreatic islets and
liver of male Goto-Kakizaki rats, a model of type 2 diabetes. PLoS One.

[11] Bisbis S, Bailbe D, Tormo MA, Picarel-Blanchot F, Derouet M, Simon J (1993). Insulin resistance in the GK rat: decreased receptor number but
normal kinase activity in liver. Am J Physiol.

[12] Matafome P, Santos-Silva D, Crisóstomo J, Rodrigues T, Rodrigues L, Sena CM (2012). Methylglyoxal causes structural and functional alterations in
adipose tissue independently of obesity. Arch Physiol Biochem.

[13] Rodrigues L, Crisóstomo J, Matafome P, Louro T, Nunes E, Seiça R (2011). Dietary restriction improves systemic and muscular oxidative
stress in type 2 diabetic Goto-Kakizaki rats. J Physiol Biochem.

[14] Kuwabara WMT, Panveloski-Costa AC, Yokota CNF, Pereira JNB, Filho JM, Torres RP (2017). Comparison of Goto-Kakizaki rats and high fat diet-induced obese
rats: are they reliable models to study type 2 diabetes
mellitus?. PLoS One.

[15] Pereira JNB, Murata GM, Sato FT, Marosti AR, Carvalho CRO, Curi R (2021). Small intestine remodeling in male Goto-Kakizaki
rats. Physiol Rep.

[16] Serdan TDA, Masi LN, Pereira JNB, Rodrigues LE, Alecrim AL, Scervino MVM (2021). Impaired brown adipose tissue is differentially modulated in
insulin-resistant obese wistar and type 2 diabetic Goto-Kakizaki
rats. Biomed Pharmacother.

[17] Marín-Peãalver JJ, Martín-Timón I, Sevillano-Collantes C, Caãizo-Gómez FJ del (2016). Update on the treatment of type 2 diabetes
mellitus. World J Diabetes.

[18] Fuchs T, Loureiro M, Both GH, Skraba HH, Costa-Casagrande TA (2017). The role of the sleeve gastrectomy and the management of type 2
diabetes. Arq Bras Cir Dig.

[19] Cornejo-Pareja I, Clemente-Postigo M, Tinahones FJ (2019). Metabolic and endocrine consequences of bariatric
surgery. Front Endocrinol (Lausanne).

[20] Li BY, Xu XY, Gan RY, Sun QC, Meng JM, Shang A (2019). Targeting gut microbiota for the prevention and management of
diabetes mellitus by dietary natural products. Foods.

[21] Kuwahara A, Matsuda K, Kuwahara Y, Asano S, Inui T, Marunaka Y (2020). Microbiota-gut-brain axis: enteroendocrine cells and the enteric
nervous system form an interface between the microbiota and the central
nervous system. Biomed Res.

[22] Singh R, Zogg H, Wei L, Bartlett A, Ghoshal UC, Rajender S (2021). Gut microbial dysbiosis in the pathogenesis of gastrointestinal
dysmotility and metabolic disorders. J Neurogastroenterol Motil.

[23] Lau E, Belda E, Picq P, Carvalho D, Ferreira-Magalhães M, Silva MM (2021). Gut microbiota changes after metabolic surgery in adult diabetic
patients with mild obesity: a randomised controlled trial. Diabetol Metab Syndr.

[24] Peng W, Huang J, Yang J, Zhang Z, Yu R, Fayyaz S (2020). Integrated 16S rRNA sequencing, metagenomics, and metabolomics to
characterize gut microbial composition, function, and fecal metabolic
phenotype in non-obese type 2 diabetic Goto-Kakizaki rats. Front Microbiol.

[B25] Kang X, Zhan L, Lu X, Song J, Zhong Y, Wang Y (2020). Characteristics of gastric microbiota in gk rats with spontaneous
diabetes: a comparative study. Diabetes Metab Syndr Obes.

[26] Péan N, Le Lay A, Brial F, Wasserscheid J, Rouch C, Vincent M (2020). Dominant gut Prevotella copri in gastrectomised non-obese
diabetic Goto-Kakizaki rats improves glucose homeostasis through enhanced
FXR signalling. Diabetologia.

[27] Adachi T, Mori C, Sakurai K, Shihara N, Tsuda K, Yasuda K (2003). Morphological changes and increased sucrase and isomaltase
activity in small intestines of insulin-deficient and type 2 diabetic
rats. Endocr J.

[28] Chen PM, Gregersen H, Zhao JB (2015). Advanced glycation end-product expression is upregulated in the
gastrointestinal tract of type 2 diabetic rats. World J Diabetes.

[29] Zhao J, Chen P, Gregersen H (2013). Morpho-mechanical intestinal remodeling in type 2 diabetic GK
rats--is it related to advanced glycation end product
formation?. J Biomech.

[30] Dou Y, Fan Y, Zhao J, Gregersen H (2006). Longitudinal residual strain and stress-strain relationship in
rat small intestine. Biomed Eng Online.

[31] Zhao JB, Frøkjær JB, Drewes AM, Ejskjaer N (2006). Upper gastrointestinal sensory-motor dysfunction in diabetes
mellitus. World J Gastroenterol.

[32] Aubé AC, Blottiàre HM, Scarpignato C, Cherbut C, Rozé C, Galmiche JP (1996). Inhibition of acetylcholine induced intestinal motility by
interleukin 1β in the rat. Gut.

[33] Yarandi SS, Srinivasan S (2014). Diabetic gastrointestinal motility disorders and the role of
enteric nervous system: current status and future directions. Neurogastroenterol Motil.

[34] Annahazi A, Schemann M (2020). The enteric nervous system: “a little brain in the
gut.”. Neuroforum.

[35] Babizhayev MA, Strokov IA, Nosikov VV, Savel'yeva EL, Sitnikov VF, Yegor EY (2015). The role of oxidative stress in diabetic neuropathy: generation
of free radical species in the glycation reaction and gene polymorphisms
encoding antioxidant enzymes to genetic susceptibility to diabetic
neuropathy in population of type i diabetic patient. Cell Biochem Biophys.

[36] Figueroa-Romero C, Sadidi M, Feldman EL (2008). Mechanisms of disease: the oxidative stress theory of diabetic
neuropathy. Rev Endocr Metab Disord.

[37] Chandrasekharan B, Anitha M, Blatt R, Shahnavaz N, Staley C, Mwangi S (2011). Colonic motor dysfunction in human diabetes is associated with
enteric neuronal loss and increased oxidative stress. Neurogastroenterol Motil.

[B38] Trevizan AR, Schneider LCL, Araújo EJA, Garcia JL, Buttow NC, Nogueira-Melo GA (2019). Acute *Toxoplasma gondii* infection alters the
number of neurons and the proportion of enteric glial cells in the duodenum
in Wistar rats. Neurogastroenterol Motil.

[39] Voukali E, Shotton HR, Lincoln J (2011). Selective responses of myenteric neurons to oxidative stress and
diabetic stimuli. Neurogastroenterol Motil.

[40] Freidin M, Bennett MVL, Kessler JA (1992). Cultured sympathetic neurons synthesize and release the cytokine
interleukin 1β. Proc Natl Acad Sci USA.

[41] Sena CM, Matafome P, Crisóstomo J, Rodrigues L, Fernandes R, Pereira P (2012). Methylglyoxal promotes oxidative stress and endothelial
dysfunction. Pharmacol Res.

[42] Rubino F, Forgione A, Cummings DE, Vix M, Gnuli D, Mingrone G (2006). The mechanism of diabetes control after gastrointestinal bypass
surgery reveals a role of the proximal small intestine in the
pathophysiology of type 2 diabetes. Ann Surg.

[43] Yu H, Song Z, Zhang H, Zheng K, Zhan J, Luo Q (2019). Duodenojejunal bypass plus sleeve gastrectomy reduces
infiltration of macrophages and secretion of TNF-α in the visceral white
adipose tissue of Goto-Kakizaki rats. Obes Surg.

[44] Rubino F, Schauer PR, Kaplan LM, Cummings DE (2010). Metabolic surgery to treat type 2 diabetes: Clinical outcomes and
mechanisms of action. Annu Rev Med.

[45] Guan W, Cui Y, Bu H, Liu J, Zhao S, Zhao Q (2020). Duodenal-jejunal exclusion surgery improves type 2 diabetes in a
rat model through regulation of early glucose metabolism. Can J Diabetes.

[B46] Camacho-Ramírez A, Prada-Oliveira JA, Ribelles-García A, Almorza-Gomar D, Pérez-Arana GM (2020). The leading role of peptide tyrosine tyrosine in glycemic control
after roux-en-y gastric bypass in rats. Obes Surg.

[47] Prada-Oliveira JA, Camacho-Ramirez A, Salas-Alvarez J, Campos-Martinez FJ, Lechuga-Sancho AM, Almorza-Gomar D (2019). GLP-1 mediated improvement of the glucose tolerance in the T2DM
GK rat model after massive jejunal resection. Ann Anat.

[48] Edholm T, Cejvan K, Abdel-Halim SM, Efendic S, Schmidt PT, Hellström PM (2009). The incretin hormones GIP and GLP-1 in diabetic rats: Effects on
insulin secretion and small bowel motility. Neurogastroenterol Motil.

[49] Svensson AM, Östenson CG, Efendic S, Jansson L (2007). Effects of glucagon-like peptide-1-(7-36)-amide on pancreatic
islet and intestinal blood perfusion in Wistar rats and diabetic GK
rats. Clin Sci (Lond).

[50] Simonsen L, Pilgaard S, Orskov C, Rosenkilde MM, Hartmann B, Holst JJ (2007). Exendin-4, but not dipeptidyl peptidase IV inhibition, increases
small intestinal mass in GK rats. Am J Physiol Gastrointest Liver Physiol.

[51] Ouyang X, Li S, Tan Y, Lin L, Yin J, Chen JDZ (2019). Intestinal electrical stimulation attenuates hyperglycemia and
prevents loss of pancreatic β cells in type 2 diabetic Goto-Kakizaki
rats. Nutr Diabetes.

[52] Forder REA, Howarth GS, Tivey DR, Hughes RJ (2007). Bacterial modulation of small intestinal goblet cells and mucin
composition during early posthatch development of poultry. Poult Sci.

[53] Shakouri MD, Iji PA, Mikkelsen LL, Cowieson AJ (2009). Intestinal function and gut microflora of broiler chickens as
influenced by cereal grains and microbial enzyme
supplementation. J Anim Physiol Anim Nutr (Berl).

[54] Kles KA, Chang EB (2006). Short-chain fatty acids impact on intestinal adaptation,
inflammation, carcinoma, and failure. Gastroenterology.

[55] Hamer HM, Jonkers D, Venema K, Vanhoutvin S, Troost FJ, Brummer RJ (2008). Review article: the role of butyrate on colonic
function. Aliment Pharmacol Ther.

[56] Kinoshita M, Suzuki Y, Saito Y (2002). Butyrate reduces colonic paracellular permeability by enhancing
PPARγ activation. Biochem Biophys Res Commun.

[57] Wang J, Chen WD, Wang YD (2020). The relationship between gut microbiota and inflammatory
diseases: the role of macrophages. Front Microbiol.

[58] Biasato I, Ferrocino I, Biasibetti E, Grego E, Dabbou S, Sereno A (2018). Modulation of intestinal microbiota, morphology and mucin
composition by dietary insect meal inclusion in free-range
chickens. BMC Vet Res.

[59] Liao X, Shao Y, Sun G, Yang Y, Zhang L, Guo Y (2020). The relationship among gut microbiota, short-chain fatty acids,
and intestinal morphology of growing and healthy broilers. Poult Sci.

[60] Yu X, Wu Z, Song Z, Zhang H, Zhan J, Yu H (2020). Single-anastomosis duodenal jejunal bypass improve glucose
metabolism by regulating gut microbiota and short-chain fatty acids in
Goto-Kakisaki rats. Front Microbiol.

[61] Kong LC, Tap J, Aron-Wisnewsky J, Pelloux V, Basdevant A, Bouillot J-L (2013). Gut microbiota after gastric bypass in human obesity: increased
richness and associations of bacterial genera with adipose tissue
genes. Am J Clin Nutr.

[B62] Furet JP, Kong LC, Tap J, Poitou C, Basdevant A, Bouillot JL (2010). Differential adaptation of human gut microbiota to bariatric
surgery-induced weight loss: links with metabolic and low-grade inflammation
markers. Diabetes.

[63] Carvalho BM, Guadagnini D, Tsukumo DML, Schenka AA, Latuf-Filho P, Vassallo J (2012). Modulation of gut microbiota by antibiotics improves insulin
signalling in high-fat fed mice. Diabetologia.

[64] Qiao Z, Han J, Feng H, Zheng H, Wu J, Gao C (2019). Fermentation products of *Paenibacillus b*ovis sp.
nov. BD3526 alleviates the symptoms of type 2 diabetes mellitus in GK
rats. Front Microbiol.

[B65] Zhao L, Chen Y, Xia F, Abudukerimu B, Zhang W, Guo Y (2018). A glucagon-like peptide-1 receptor agonist lowers weight by
modulating the structure of gut microbiota. Front Endocrinol (Lausanne).

[B66] Zhang C, Wu W, Xin X, Li X, Liu D (2019). Extract of ice plant (Mesembryanthemum crystallinum) ameliorates
hyperglycemia and modulates the gut microbiota composition in type 2
diabetic Goto-Kakizaki rats. Food Funct.

[B67] Zhao J, Li Y, Sun M, Xin L, Wang T, Wei L (2019). The Chinese herbal formula Shenzhu Tiaopi granule results in
metabolic improvement in type 2 diabetic rats by modulating the gut
microbiota. Evid Based Complement Alternat Med.

[B68] Zhao F, Liu Q, Cao J, Xu Y, Pei Z, Fan H (2020). A sea cucumber (*Holothuria leucospilota*)
polysaccharide improves the gut microbiome to alleviate the symptoms of type
2 diabetes mellitus in Goto-Kakizaki rats. Food Chem Toxicol.

[B69] Tan C, Zheng Z, Wan X, Cao J, Wei R, Duan J (2021). The role of gut microbiota and amino metabolism in the effects of
improvement of islet β-cell function after modified jejunoileal
bypass. Sci Rep.

[70] Zhao JD, Li Y, Sun M, Yu CJ, Li JY, Wang SH (2021). Effect of berberine on hyperglycaemia and gut microbiota
composition in type 2 diabetic Goto-Kakizaki rats. World J Gastroenterol.

[71] Collins J, Borojevic R, Verdu EF, Huizinga JD, Ratcliffe EM (2014). Intestinal microbiota influence the early postnatal development
of the enteric nervous system. Neurogastroenterol Motil.

[72] Knauf C, Abot A, Wemelle E, Cani PD (2020). Targeting the enteric nervous system to treat metabolic
disorders? “Enterosynes” as therapeutic gut factors. Neuroendocrinology.

[73] Abot A, Cani PD, Knauf C (2018). Impact of intestinal peptides on the enteric nervous system:
novel approaches to control glucose metabolism and food
intake. Front Endocrinol (Lausanne).

[74] Yissachar N (2020). Ménage è trois: regulation of host immunity by enteric
neuro-immune-microbiota cross talks. Curr Opin Neurobiol.

[75] Carabotti M, Scirocco A, Maselli MA, Severi C (2015). The gut-brain axis: Interactions between enteric microbiota,
central and enteric nervous systems. Ann Gastroenterol.

[76] Fournel A, Drougard A, Duparc T, Marlin A, Brierley SM, Castro J (2017). Apelin targets gut contraction to control glucose metabolism via
the brain. Gut.

